# Interaction of two parenchyma ray types regulates redwood heartwood deposition

**DOI:** 10.1038/s41598-026-42938-6

**Published:** 2026-03-31

**Authors:** Alana R. O. Chin, Stephen C. Sillett, Omar Laín, Klara Voggeneder, Jessica Orozco, Zane Moore, Marc Bast, N. Dilworth Parkinson, Paula Guzmán-Delgado

**Affiliations:** 1 Department of Biological Sciences, Cal Poly Humboldt, Arcata, CA USA; 2 Department of Forestry, Fire, and Rangeland Management, Cal Poly Humboldt, Arcata, CA USA; 3 Plant Ecology Group, Institute of Integrative Biology (IBZ), ETH-Zürich, Zürich, ZH Switzerland; 4 Earth and Planetary Science Department, Stanford University, Stanford, CA USA; 5 Menlo School, Atherton, CA USA; 6 US Department of Energy, LBNL Advanced Light Source, Berkeley, CA USA; 7 Department of Plant Science, UC Davis, Davis, CA USA

**Keywords:** Heartwood extractives, MicroCT, Parenchyma ray, Redwood, *Sequoia sempervirens*, Wood anatomy, Wood density, X-ray densitometry, Ecology, Ecology, Plant sciences

## Abstract

Part of the longevity strategy of trees is the deposition of extractives, biocidal compounds within heartwood that slow decay long after tree death and are thus an important determinant of carbon residency time. The process of heartwood deposition is regulated by parenchyma rays, living tissues that store carbohydrates, are the sites of biosynthesis, and contain substantial quantities of extractives within mature heartwood. We sought to explore and predict heartwood deposition across the full axial gradient in *Sequoia sempervirens* by incorporating the plasticity of sapwood parenchyma rays into our models and testing the value of separating them into two size classes. We used synchrotron-based X-ray tomographic microscopy (microCT) for anatomical measurements and novel tissue-level densitometry of wood samples collected from up to 100 m above ground. We found that *Sequoia* has two distinct ray types, and the interaction of these short and tall rays strongly predicts variation in the extractive content of heartwood. Environmental context was crucial; separate anatomy-based models were needed for never-logged primary and recovering secondary *Sequoia* forests in the north and primary forests on the southern margin of the species’ range. Incorporating ray structure and distribution into future work on heartwood extractives could provide tools to quantify and monitor heartwood responses of *Sequoia* forests to management and climate based on the phenotypic plasticity of rays.

**Supplementary Information:**

The online version contains supplementary material available at 10.1038/s41598-026-42938-6.

## Introduction

Trees deposit secondary metabolites (extractives) in heartwood that promote their longevity and influence forest carbon residency-time by increasing wood decay resistance^1–5^. The process of heartwood formation relies on the living ray parenchyma that synthesize and store extractives during the conversion of sapwood to heartwood^3,6–8^. Rays, files of parenchyma cells that run ribbon-like from the phloem toward the pith between non-living wood tracheids, transport substances radially within the trunk and branches and store reserve carbohydrates in the sapwood (Jeffery 1917)^9,10^. Rays are responsible for the storage of nearly all wood-level non-structural carbohydrates in living cells as well as deposition of heartwood extractives in non-living cells. The conversion of living sapwood to dead heartwood comes with an increase in wood density due to the mass of often colorful extractives held primarily in rays^6–8^. Coast redwood (*Sequoia sempervirens*) has deep red heartwood up to 25% denser than light-colored sapwood, contributing to longevity over 2000 years, development of giant trees, and persistence of fallen trunks that enable redwood forests to accumulate more biomass than any other terrestrial system (Sillett et al*.* 2020). The estimated mass of heartwood extractives in *Sequoia* also varies axially along the trunk (highest toward treetop), extractives are richer in taller primary versus shorter secondary forests, and in dry southern (< 37° latitude) compared to wet northern forests (> 40° latitude) for trees of similar height, reflecting tradeoffs among growth efficiency, heartwood investment, and climatic sensitivity (Sillett et al*.* 2020, 2022, 2025).

**Fig. 1:**
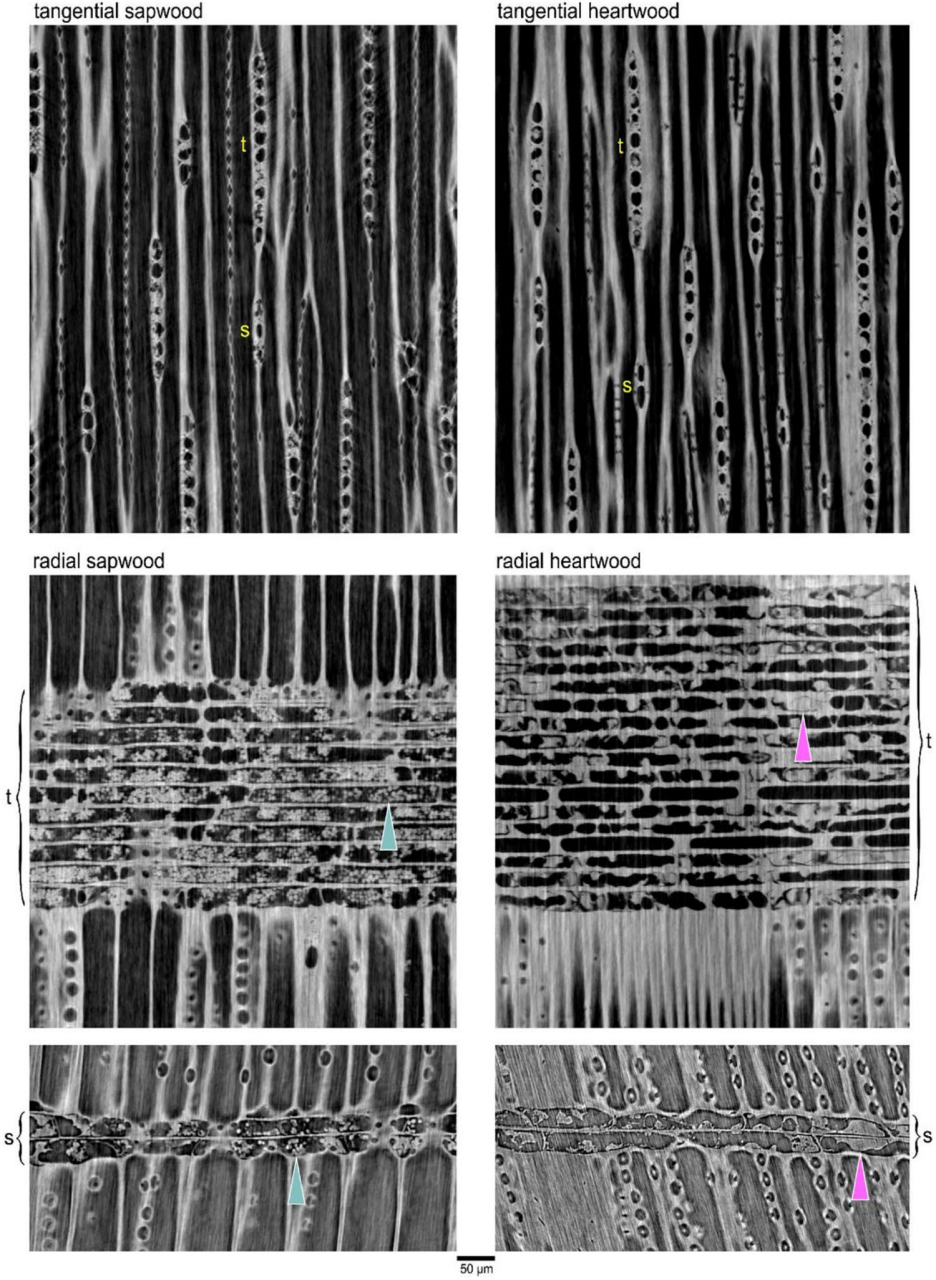
Short (s) and tall (t) parenchyma rays of *Sequoia*. Shown in tangential and radial planes, starch storing amyloplasts, seen as small balls (examples at blue arrows), are visible within both ray types in living sapwood, while phenolic deposits (examples at pink arrows) can be seen instead within dead rays of heartwood.

Although endogenous and exogenous regulators of heartwood formation, such as cambial age and climate, are not entirely clear, parenchyma rays transport and store the carbohydrates that fuel production of heartwood extractives and are the primary site of their deposition^7,8^, Fig. 1). Individual parenchyma rays are produced by a dedicated set of cells in the cambium and traverse years of wood staying the same height, linking the sugar-filled phloem near the youngest sapwood to the heartwood transition zone^10,11^. The initiation and termination of rays accordingly has long-term influences on wood physiology. However, rays are plastic and respond to climatic signals such as precipitation and temperature (Weimann et al*.* 1997, Godfrey et al*.* 2021^12–14^, implying that links between climate and heartwood may be in part mediated by this anatomical responsiveness. Can the anatomy of rays in recent sapwood predict the quantity of extractives that will be deposited in the heartwood? If so, predicting variation in extractive content may reveal mechanisms of their formation and contribute to understanding how site conditions or management activities may influence heartwood formation.

The objective of our study is to relate *Sequoia* ray anatomy to the quantity of heartwood extractives. We use differences in physical density (dry mass ÷ fresh volume) between paired samples of heartwood and sapwood to infer the amount of extractives because this accessible metric has proven useful in the development of allometric equations for estimating *Sequoia* biomass and quantifying heartwood investment (Sillett et al. 2020, 2022, 2025). Since preliminary observations indicated two distinct ray morphotypes (*i.e.,* short and tall rays with either few or many cells per file in tangential view, respectively), we explore their distinctness and functional significance here (Fig. 1). Using synchrotron-based X-ray microscopy (microCT) scans, we investigate the structure and X-ray absorbance (associated with physical density) of rays in sapwood and heartwood samples. Trunk core samples come from 10–100 m above the ground and represent northern primary, southern primary, and northern secondary *Sequoia* forests. We use fully convolutional network models to discern ray types and predict heartwood deposition using ray anatomy. We hypothesize that the two ray types are distinctly different in structure and function and that their size and distribution in recent sapwood influences the quantity of heartwood extractives. If the ray types are distinct, then they should have different distributions, different physical densities, be consistently recognizable using machine learning tools, and better predict heartwood deposition collectively or individually than when pooled into total rays. The ability to use current-year sapwood structure to predict heartwood extractive content would allow traditional histological methods to be employed in rapid assessment of the impacts of disturbances (*e.g*., drought) and experimental treatments (*e.g*., silviculture, rain exclusion) on forest carbon storage.

## Methods

### Study trees and samples

A rangewide analysis of biomass, productivity, and carbon storage in tall primary (unlogged) and secondary (post-logging regeneration) *Sequoia* forests yielded a set of trunk cores from which we randomly sampled^15^. Our samples were collected from 20 sites (under permits granted to S. Sillett) managed by California State Parks, US Forest Service, City of Arcata, Land Trust of Napa County, Marin County Parks, East Bay Regional Park District, Santa Lucia Conservancy, Bureau of Land Management, University of CA, and private landowners (details in Supplemental Table 1, and map in SupplementalFig. 1). The diversity of sites in our existing trunk core collection (see^15^) provided a well-replicated set of paired heartwood and sapwood samples ideal for this study without the need to collect new plant material. No formal vouchers are deposited due to the ease of species identification.

Excluding the sapwood-heartwood transition zone by ~ 1 cm on each side, paired samples separated most of the recent sapwood from 5–10 cm of heartwood per core. Sealed in airtight tubes immediately after collection, samples were individually measured for fresh volume (via Archimedes method) and dry mass (101 °C for 48 h to remove bound water and reach constant mass) to obtain dry-mass-to-fresh-volume ratios (precision: 0.001 g & 1 mm^3^). For each forest type, we randomly selected 3–4 replicates per 10-m height interval from the set of trunk core-samples (Table 1). Each individual tree provided a single sapwood-heartwood pair for a single height. In northern primary forests (> 40° latitude), we used 36 trees (69–108 m tall, 227–1753 yr old) sampled from 10–90 m; in southern primary forests (< 37° latitude), we used 21 trees (64–93 m tall, 177–1286 yr old) sampled from 10–70 m; and in northern secondary forests (> 40° latitude), we used 24 trees (58–76 m tall, 97–150 yr old) sampled from 10–60 m (Supplemental Table 1).

**Table 1:**
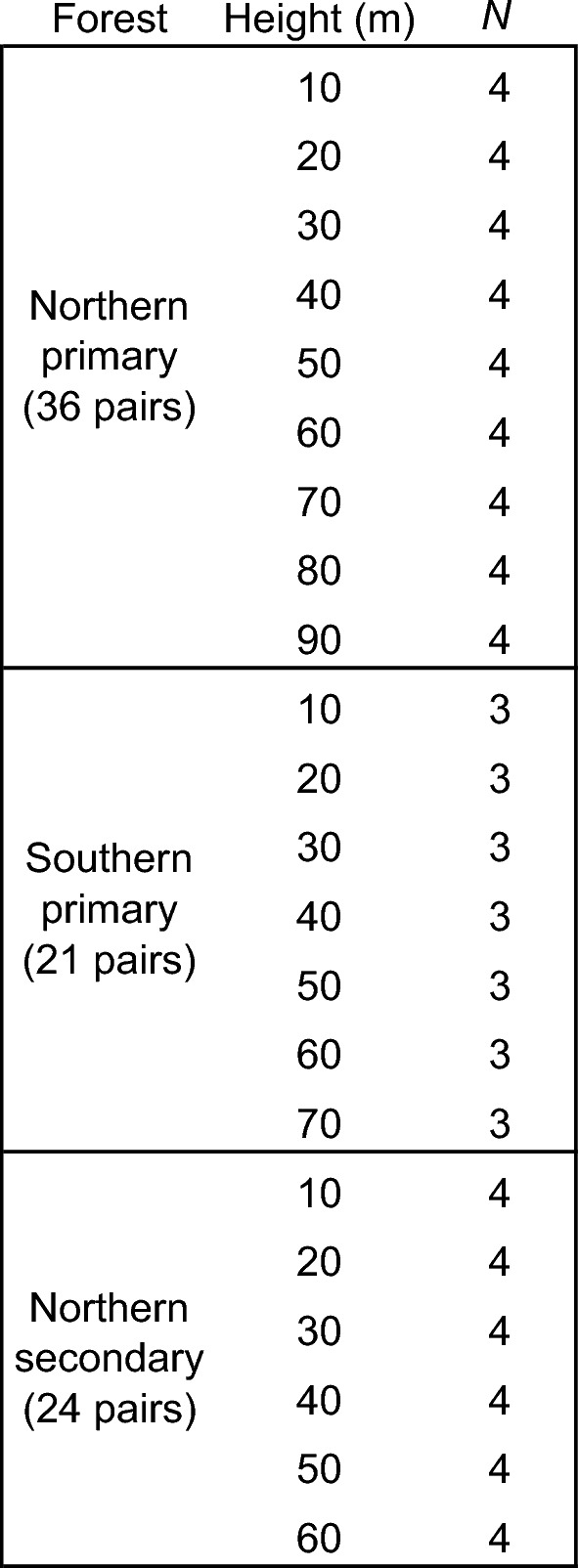
Sample size and height distribution.

### MicroCT

Wood cores were scanned using X-ray tomographic microscopy (microCT) at the US Advanced Light Source (ALS) beamline 8.3.2. The ALS at Lawrence Berkeley National Laboratory (Berkeley CA, USA) is a synchrotronic particle accelerator capable of producing the X-rays needed for our work. We obtained CT scans for 162 core segments from the 81 study trees using a beam energy of 22 keV with 10X optical magnification and yielding a pixel resolution of 0.56 µm. Scans were reconstructed using ALS scripts (TomoPy) to preserve X-ray absorbance, that is, contrast was not manipulated, and no pseudo-phase techniques were used. All datasets were scaled the same such that brightness of the background is zero in the 8-bit scale, thereby normalizing X-ray absorbance against tiny fluctuations in beam intensity among scans. During reconstruction, all background (air) voxels were set to a brightness of zero to relativize X-ray absorbance.

A second set of microCT scans was made from cores collected at 10-m intervals from 10–100 m (20 CT scans) in a primary forest tree (107 m tall, 874 yr old) at the Swiss Light Source (Villagen, Switzerland) TOMCAT beamline. These scans were made with the intention of better observing wood anatomy and cellular contents at the expense of X-ray absorption interpretability. High image sharpness, greater magnification, and different reconstruction choices complemented the density-focused work done at ALS. We used a beam energy of 21 keV and an optical magnification of 20X to produce scans with a pixel resolution of 0.33 µm. To enhance visual clarity, we used pseudo phase-contrast Paganin reconstruction^16^ and adjusted the contrast of individual images to avoid white saturation. These higher resolution scans were used for visualization of ray structure and contents across the vertical gradient (Fig. 1).

### Analysis of anatomy and X-ray absorbance

Absorption of X-rays increases with electron density and is reflected in the brightness of the resulting CT scan, where brighter pixels contained more electrons. Electron density can serve as a proxy for physical density, especially in the case of wood, where the cells (polysaccharide based) and heartwood extractives (phenolics) both consist almost entirely of C, H, O, N, and P and mostly lack metals and high-density crystals. These convenient features allowed us to assume a linear relationship between brightness and physical density of the cells, a similar concept to the widely used wood densitometer but with cell-level resolution. Within each scan, we selected the first central slice that was clear of artefacts and annotated every pixel with colors indicating 5 categories— latewood, earlywood, short rays, tall rays, background— with background being important to include for removal of occasional cracks. In total, our annotation process involved manually coloring > 1.7B pixels. Annotated slices were used to select regions of interest (ROI) representing each tissue class in Fiji so that ROI could be used to obtain 256-bin brightness histograms for each of our 4 wood tissue classes per CT scan (a total of 648 histograms). We computed the mean brightness value of the full tissue histogram, including all negative-values (shadow artefacts appearing less dense than air) and corresponding improbably-bright pixels to avoid bias in excluding artefacts. Mean brightness (*i.e*., relative X-ray absorbance) served as a tissue-level physical density proxy. Due to large differences in variance, brightness was compared among tissues and scans with permutation-based asymptotic general independence tests in the R package coin. Using the same tissue ROI, we measured short and tall ray size, maximum length, and density (Table 2) for use in modeling heartwood extractives. We also looked for relationships between these ray anatomical metrics and sample height, age of the local vascular cambium, and other tree features such as cambium surface area, wood volume, and trunk diameter. We made pair-wise comparisons of anatomical features between forest types using permutation-based two-tailed Asymptotic General Independence tests with the *p*-value Bonferroni corrected for the total number of comparisons.

**Table 2:**
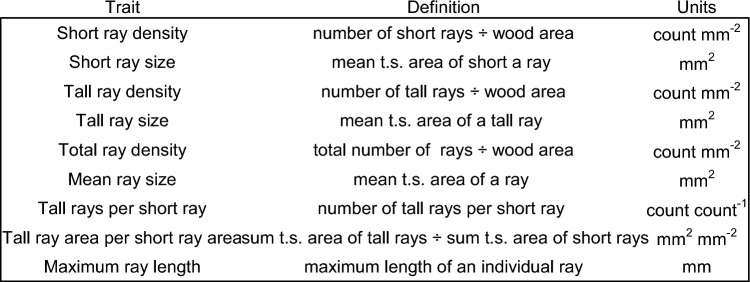
Anatomical traits of *Sequoia sempervirens* trunk wood. *t.s.* = *tangential section*.

### Computer recognition of ray types

**Fig. 2:**
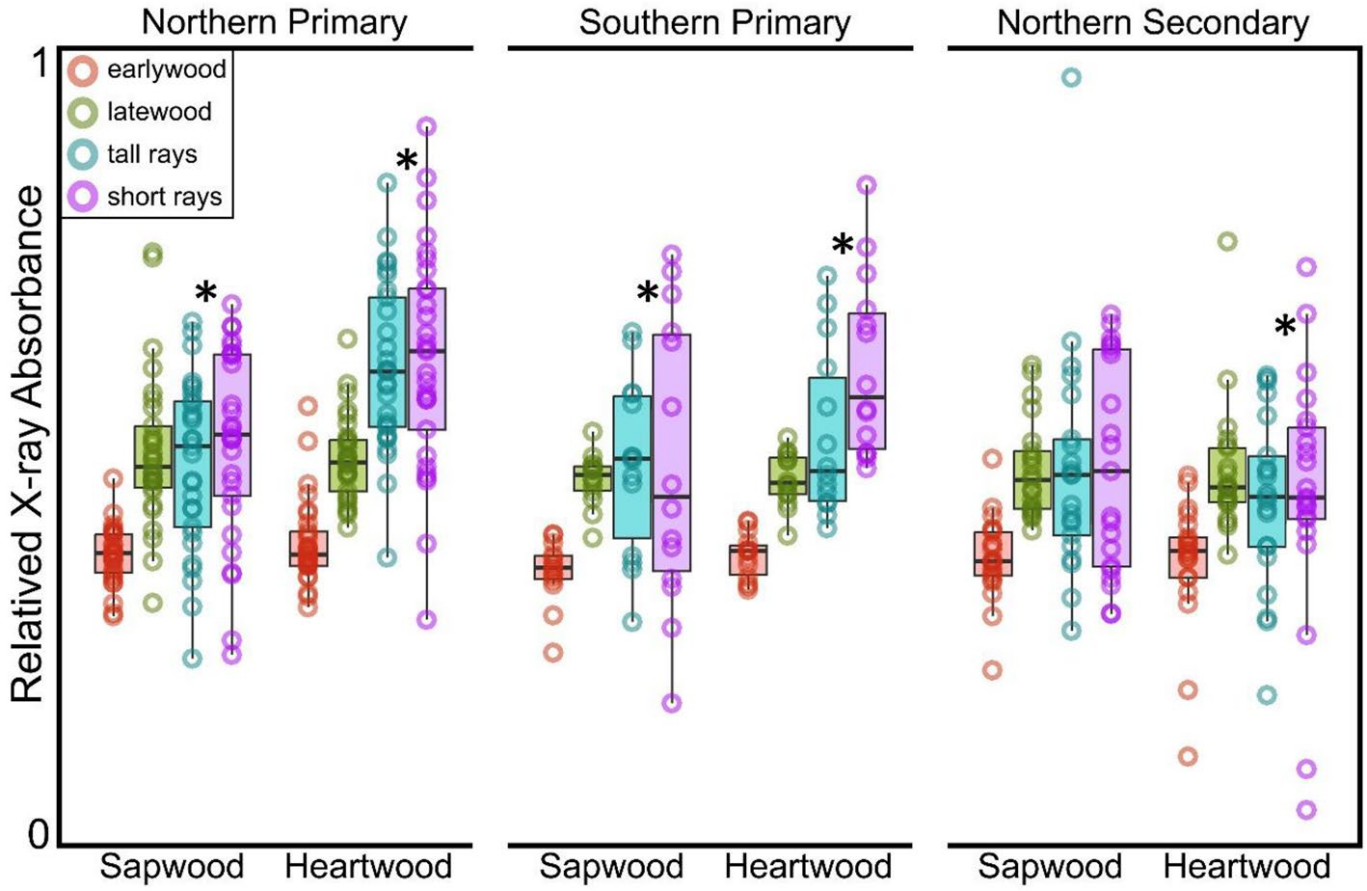
Relative X-Ray absorbance of *Sequoia* wood tissue types at 0.56-μm resolution. Greater X-Ray absorbance (measured as pixel brightness) is indicative of greater physical density. Asterisks indicate significant differences in X-Ray absorbance between ray types within that wood and forest type. Note that only in northern secondary forest sapwood did short and tall rays have equivalent absorbance signatures. Box plots indicate quartile ranges.

The rays in *Sequoia* are mostly only one cell wide (uniseriate) with the occasional biseriate or partially biseriate ray. Two types of uniseriate ray, short and tall, as described and measured here (Fig. 1.), have not, to our knowledge, previously been mentioned in the *Sequoia* literature. However visually distinct they appeared to us (Figs. 1 & 2), we wanted further evidence of distinctiveness and turned to machine-based visual discrimination as a means of verification. Fully Convolutional Network Models (FCN models) have proven to be extraordinarily good at semantic segmentation of microCT images where the goal is typically to make 3D measurements^17^. We follow the FCN modeling framework developed by^17^ for semantic segmentation of microCT images. In our case, we were primarily interested in recognition of rays as short and tall morphotypes, applying the logic that if the model agrees with our classification of unfamiliar images, then two consistently distinct types exist. We trained our model for sapwood only, using 77 of 81 annotated sapwood slices to train the model and 4 to test it with all 4 coming from scans to which the model was naïve. Annotated slices included color indication of 5 the categories of pixel identity (short rays, tall rays, latewood, earlywood, background) that the model was trained to recognize. For computational efficiency, images were scaled down to 80% of their original size. Using a validation fraction of 0.05, we created 5 models that each ran for 200 epochs. We selected the best of the five models based on its ability to distinguish rays. Model-annotated slices of the 4 test scans were then compared to hand-annotated slices to assess recognition accuracy on the ray level based on color assigned to the object core for 1916 rays in the test scans.

### Modeling heartwood extractives

The goal of this study was to predict heartwood extractive content using sapwood anatomy, relying only on features that could be measured with traditional histological tools. As a predictand, we used heartwood extractive content computed as heartwood density – sapwood density per sample pair, retaining only those with inferred extractive content > 0 and < 100 kg m^−3^ to avoid several extreme values too distant from the rest not to have undue leverage (*N* = 67). Because we had a large number of collinear traits (Table 2) and a fairly small sample size, we used LASSO (least absolute shrinkage and selection operator) regression to select variables and regularize their coefficients. We fit a unique model for each forest type to confirm roles of the 2 ray types. A regularization hyperparameter λ was selected for each model using fivefold cross validation to find the optimal λ based on the lowest MSE. Models were fit with the R package glmnet using penalized maximum likelihood as the estimator. To assess goodness of fit, *R*^2^ was computed from predicted and observed values.

## Results

### Anatomy and X-ray absorbance

**Fig. 3:**
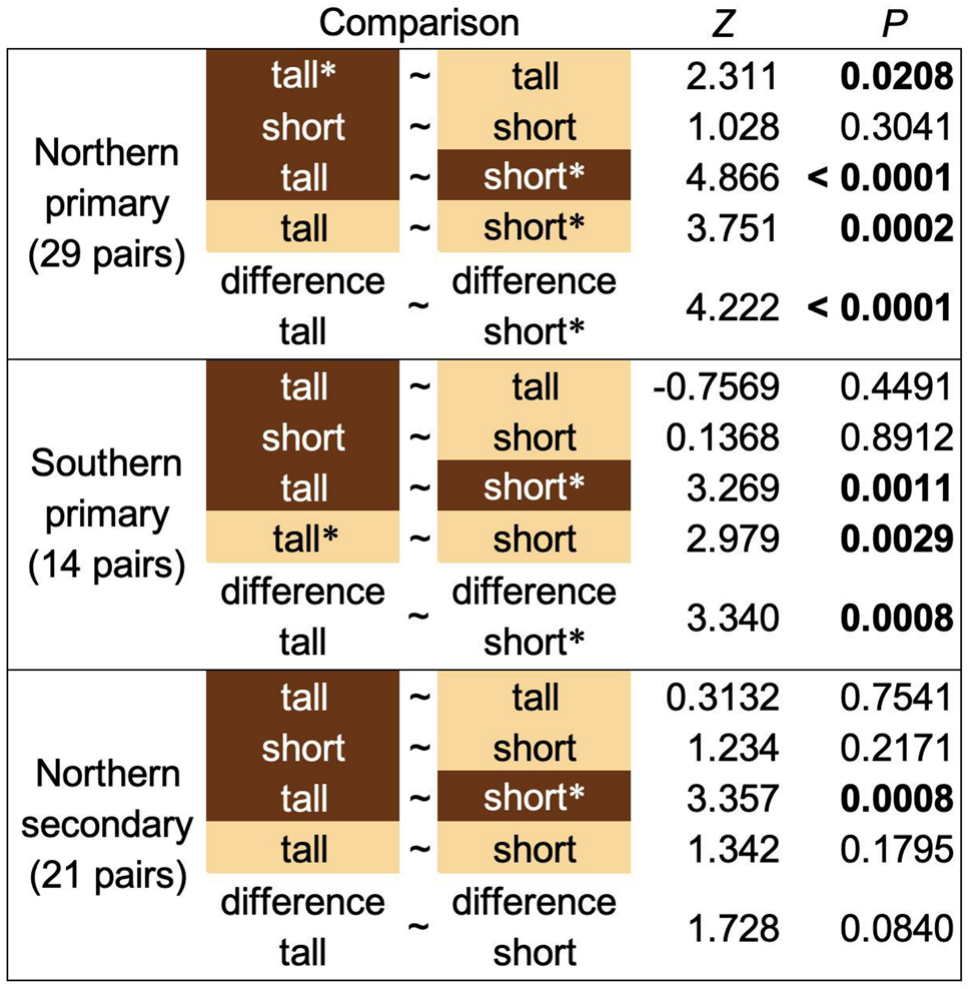
Differences in X-Ray absorbance in the short and tall parenchyma rays of *Sequoia* wood at 0.56-μm resolution. Heartwood rays are indicated by a brown color while sapwood rays are tan. Comparisons noted as “difference” refer to heartwood minus sapwood X-ray absorbance. Asterisks indicate the ray type with greater absorbance for each significant comparison. Bonferroni-corrected *P*-values are from asymptotic general independence tests, where Z is the test statistic. Significant absorption comparisons are shown in bold.

In all samples from primary forests, short rays had greater physical density (X-ray absorbance) in heartwood than tall rays, and a greater density increase from sapwood to heartwood suggesting that short rays accumulate more extractives (Fig. 2). Conversely, in secondary forests, a dramatic increase in ray physical density due to heartwood extractives was not indicated by X-ray absorbance (Fig. 3). Indeed, tall rays in the heartwood were slightly, but significantly, *less physically dense* than those in sapwood (Figs. 2**&3**), presumably due to the mass of heartwood extractives being lower than the mass of consumed amyloplasts that are absent in heartwood (Fig. 1). Within heartwood, short and tall rays did not have the same physical densities, suggesting different roles regardless of differences associated with forest type (Fig. 2). Northern primary forests exhibited a dramatic increase in tall ray physical density that was not apparent in the other forest types (Fig. 3). While latewood was always denser than earlywood, X-ray absorbance did not indicate a significant physical density difference between sapwood and heartwood when considering tracheids only, which was true in both latewood and earlywood for all forest types (Fig. 2). Tracheids within the heartwood of these cores appeared stained even to the naked eye, and their fluorescent properties indicated the presence of heartwood-associated phenolics (A. Chin, pers. obs.). The inability of our microCT method to detect extractive presence as electron density in tracheids does not imply that they are absent, but this does suggest that rays contain far more extractives per unit volume than do tracheids.

Across forest types, short and tall rays occurred in roughly equal proportion to each other, perhaps further indication of synergistic roles (Table 3). We did not find any convincing relationships between ray anatomy and either sample height or features of the individual trees when considering all forest types, but trees in secondary forests had traces of height-associated trends in ray anatomy that were lacking from primary forests in the north or south (Table 3). Relationships between cambial age and ray anatomy were inconsistent with primary forest samples in many cases showing an opposite-direction relationship from secondary forest samples. Contrasting responses to the age of the local vascular cambium were especially notable in the length of tall rays, which had a positive relationship to cambial age in secondary forests and a negative relationship in primary forests (Table 3). Ray anatomy differed among forest types with significantly larger tall rays in southern primary forests and more rays per area (*i.e*., greater spatial density) as well as more tall rays per short ray in northern secondary forests (Table 3)**.**

**Table 3:**
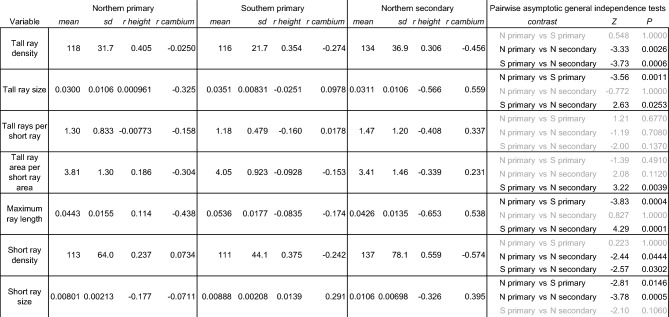
Anatomical traits of *Sequoia sempervirens* trunk wood, including means, standard deviations (sd), and Pearson correlations (*r*) with sample height and local cambium age split by forest type. Tall and short ray density is spatial, referring to the number of rays per mm^2^, not physical density as related to X-ray absorbance. Pairwise contrasts between forest types are from asymptotic general independence tests (Z is the test statistic) with *p*-values Bonferroni-corrected for the multiple comparisons. Nonsignificant differences are indicated in gray.

### Computer recognition of ray types

**Fig. 4:**
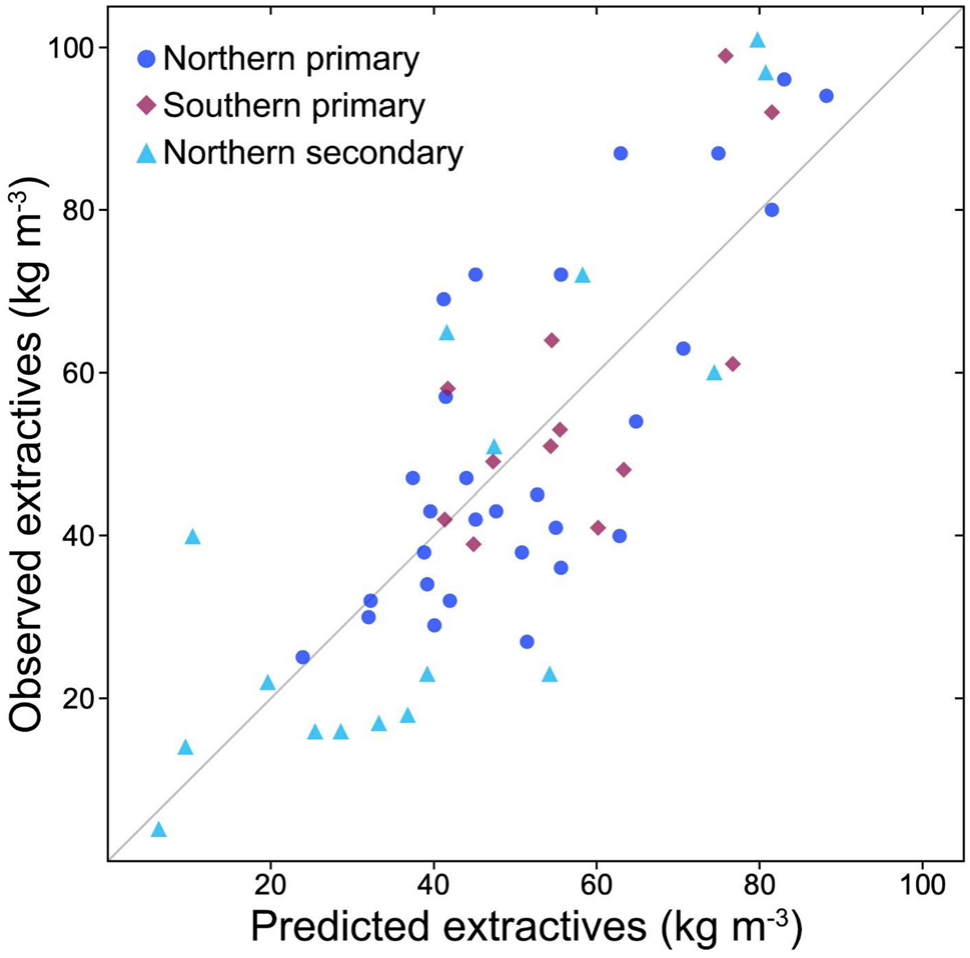
Short and tall rays in sapwood predict heartwood extractives in *Sequoia*. LASSO models showing predicted vs. observed values for extractives inferred from differences between heartwood and sapwood densities of paired samples (kg m^−3^). Each forest type had an individual sapwood-anatomy based model, and in all three cases the best model contained both ray types. Northern primary *R*^2^ = 0.58, northern secondary *R*^2^ = 0.70, southern primary *R*^2^ = 0.53.

Our FCN model generally discriminated ray type on an object-level (having > 2/3 central pixels correct), correctly classifying 329 of 372 short rays (88.4%) and 578 of 586 tall rays (98.6%) present in the test images (Fig. 4). On a pixel-level, model performance was strong but pixels of short rays (precision = 0.86, recall = 0.43, accuracy = 0.99) were more likely to be missed than those of tall rays (precision = 0.92, recall = 0.74, accuracy = 0.82) where precision was high. More than 4X as many pixels belonged to tall rays, making accuracy (true negatives) naturally higher for short rays because chance mistakes were more likely to miss a short ray than a tall ray. Most often the lower precision for short rays was because the borders of short rays were frequently misclassified as tall rays (Fig. 4), and pit fields between tracheids were sometimes misclassified as both ray types with classification as “tall rays” being ~ 29% more likely but still leading to a low recall score for the less represented short rays. The small size of many of these pit fields classed as tall rays suggests that the model is using features beyond size in segmentation.

### Modeling heartwood deposits

**Fig. 5:**
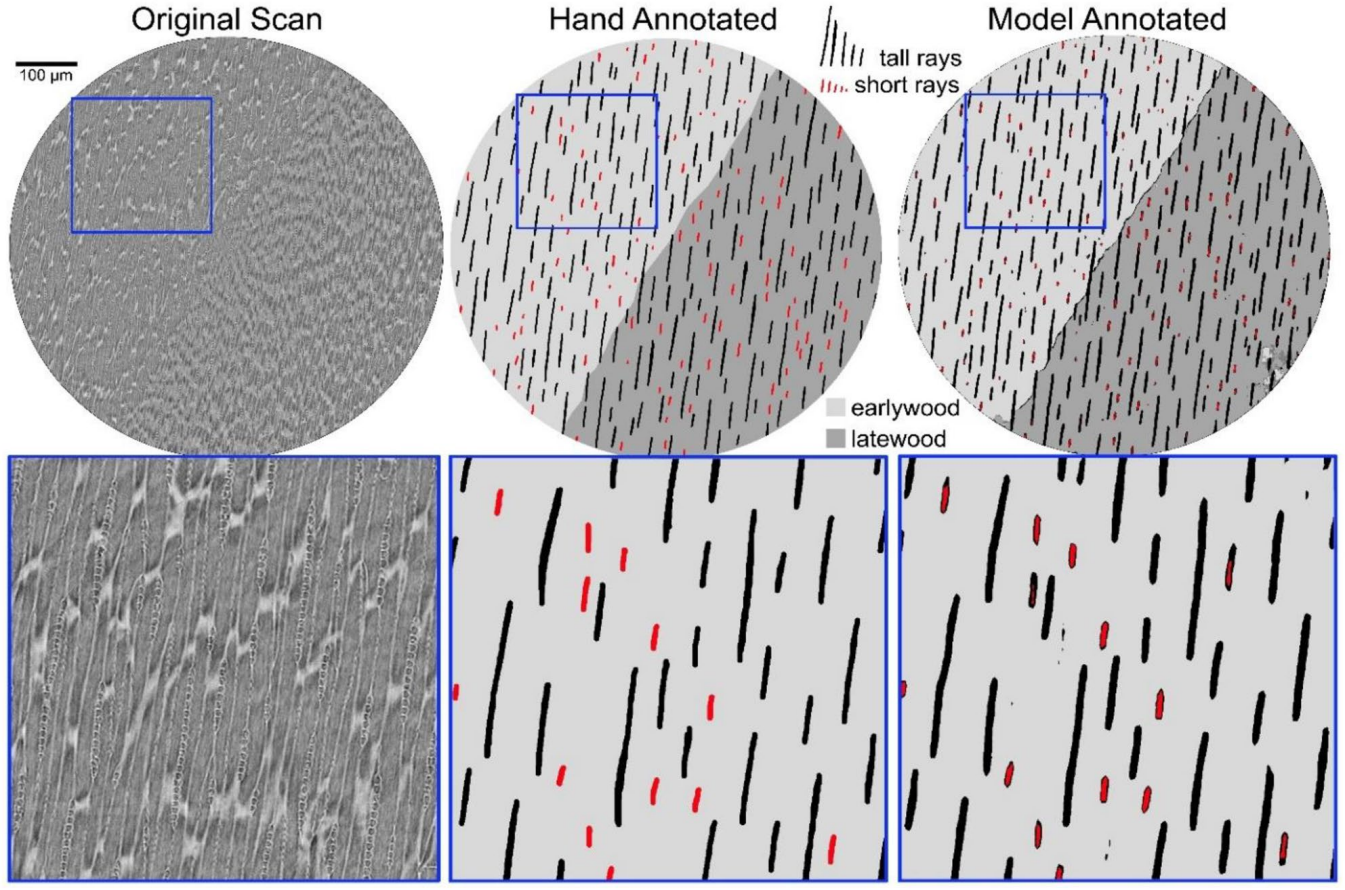
Fully convolutional network models generally succeed in differentiating between short and tall rays. On original raw scans (left), reconstructed to preserve density information, we manually annotated every pixel (~ 1.7 B) to classify earlywood (light grey), latewood (dark grey), short rays (red), tall rays (black), and background. These images were used for model training and to extract anatomy data. The resulting model was capable of semantic segmentation of scans on which it had not been trained (center), outputting images correctly classifying nearly all rays (right). Blue square cutouts from each scan are shown 3X larger in boxes at bottom.

Differences between heartwood and sapwood densities of paired samples were related to sapwood ray anatomy (traits in Table 2) in all three forest types, and in all cases the top model included both individual ray types but never total ray area and density (Table 4). Indeed, models with only total ray metrics and no separation of short and tall types failed to converge. For all three forest types, short ray density was by far the most important predictor with a universally positive relationship to heartwood extractive content (Table 4). This key trait had a consistent interaction with tall ray size, maximum length, and density (Fig. 5**,** Table 4). We were able to predict most of the variation in heartwood extractive content in northern secondary forests (*R*^2^ = 0.698), northern primary forests (*R*^2^ = 0.581), and southern primary forests (*R*^2^ = 0.529) using sapwood ray anatomy alone (Table 4**, **Fig. 4). We repeated these models using heartwood anatomy data, and although the same basic parameter structure remained, they did not fit as well as the models using sapwood anatomy, suggesting ray storage space was less important than current control of deposition. In all cases, sample height (which had no clear relationships to wood anatomy) improved these models, but exploring this established relationship was not a goal of this study.

**Table 4:**
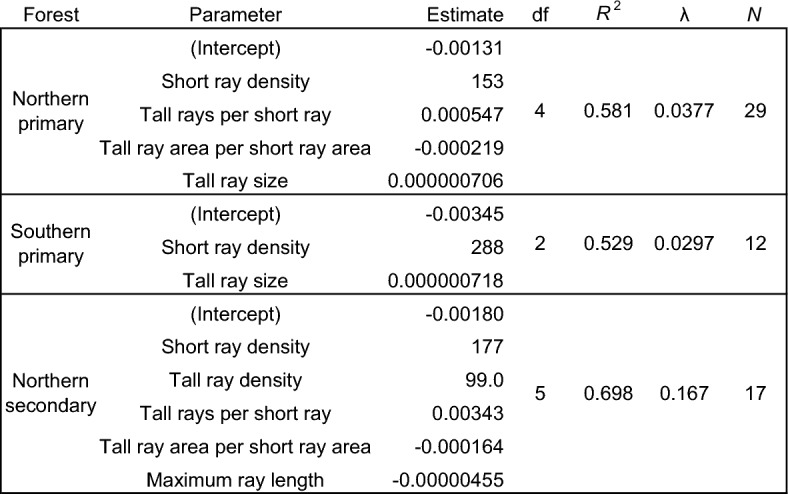
Results of LASSO regression models predicting *Sequoia sempervirens* heartwood deposits from wood anatomy. The unique regularization coefficient (λ) for each model was selected using a fivefold cross validation procedure.

## Discussion

*Sequoia* possesses two distinct types of living parenchyma rays in sapwood with interacting and apparently different roles regulating heartwood extractive content in primary and secondary forests (Fig. 5). Short and tall ray types (Fig. 1) warrant consideration as separate entities due to the combined evidence of their ability to predict heartwood extractive content when considered separately (Fig. 5), their density differences as indicated by X-ray absorbance (Fig. 3), and their reliable recognition by a fully convolutional network model (Fig. 4). While two types of uniseriate rays in conifers have not been previously described outside of the developmental literature (noted in *Pinus*, see^10,11^), we do not think they are unique to either *Sequoia, Pinus*, or their families, but rather widespread based on numerous wood images available in classic conifer anatomy books^18–21^. Explorations of ray phenotypic plasticity and physiology should thus incorporate metrics describing these ray types as independently varying and functioning tissues.

Our sapwood ray anatomy models using both ray types explain over half of the variation in heartwood extractive content in all three forest types (Fig. 5**, **Table 4). While noise in our density-based heartwood extractive metric due to variation in sapwood non-structural carbohydrate content (see abundant amyloplasts in Fig. 1) is not considered here, the success of our models indicates a biologically meaningful connection between the structure of active sapwood and the deposition of extractives in *Sequoia* heartwood. Within sapwood, short and tall rays have similar X-ray-indicated density. However, within the heartwood, short rays are higher density than tall rays in all three forest types. Short rays being denser than tall rays in heartwood is the only significant heartwood/sapwood density difference in secondary forests detectable with our microCT X-ray densitometry approach (Figs. 2, 3). The number of short rays per tangential wood area is the best predictor of extractive content in all models (Fig. 5), but total percent parenchyma and the number of all parenchyma rays per area are not useful predictors in any model. The connection between ray type and heartwood extractive content is important for two main reasons. First, it points towards unexplored physiological phenomena involving functional differences in the tall and short ray types. Second, it suggests we can use current-year variation in traits like the number of short rays per area to assess how present conditions shape future heartwood deposition. Tracking pre- and post-treatment ray anatomy may eventually help bring impacts on future heartwood deposition into the assessment toolkit of forest managers. Ray lines can persist for years to decades, merging and splitting to maintain parenchyma continuity all the way from cambium to heartwood^10^. The phenotypic plasticity of wood in response to climatic factors or silvicultural treatment could thus control heartwood deposition in a long-term manner.

At the cambial level or very early in cell differentiation, the size of parenchyma rays and total ray fraction of wood change as new ray lines are initiated and old ones continued or terminated, and these shifts in wood anatomy respond to climate in individuals and vary along ecological gradients within and among species, Godfrey et al*.* 2021^12,22–26^. Factors increasing either ray numbers or percent parenchyma include cold (numbers^27^:), heat (percent: Weimann et al*.* 1997)^12^ and high water availability^12,13^, Godfrey et al*.* 2021) with conditions before and during ray formation playing a role^14^. The timing of water availability is key. Continuity of existing rays and initiation of new ray lines have both been linked to seasonal timing of rainfall in temperate conifers, especially the prior winter and fall^24,26^. Winter rainfall seems to lead to fewer new rays but increased retention of established ray lines in individual trees, while a wet May increases the total percent ray parenchyma in wood^24^. Lagged seasonal effects suggest a relationship to carbohydrate reserve status and perhaps current or projected storage needs. Even longer delayed anatomical responses occur in mature *Pinus sylvestris* trees that waited several years before increasing ray area fraction (percent parenchyma) in response to experimental irrigation^13^. The critical role of rays in whole-plant carbohydrate storage may be linked to seasonal and legacy effects on ray development, pinning long-term wood function on phenotypic plasticity. Further work is needed across conifers with uniseriate parenchyma rays to understand how the balance of short and tall rays shifts with climatic factors and is influenced by carbohydrate availability and cambial activity.

Although ray fractions do not necessarily predict non-structural carbohydrate concentration within species Godfrey et al*.* 2021^13,27^, they predict nonstructural carbohydrate concentrations among species and across clades (Morris et al*.* 2015)^28^. Ray area has been used as a proxy for metabolic activity and tree vigor^29^, and in our study the total percent ray parenchyma was greatest in northern secondary forests, followed by southern primary forests with northern primary forests having the least total ray fraction in their wood— a ranking reflective of observed differences in growth efficiency among these forest types^15^. A greater proportion of rays is likely associated with an enhanced capacity for relatively rapid stress responses, for example, through the mobilization of non-structural carbohydrates from inner stem regions to other stem tissues or organs (Dietze et al*.* 2013)^30^. Within species, greater wood area is taken up by parenchyma rays on the wet margins of their range (Godfrey et al*.* 2021), and globally, wet tropical species have the greatest ray areas (Morris et al*.* 2015). In *Sequoia*, we find informative anatomical models predicting heartwood extractive content for primary forests on both the north and south ends of the species range (Fig. 5**, **Table 4), but these models only are successful when looking at forest regions separately and not when pooled, perhaps due to functional impacts of the significantly larger size of both short and tall rays in southern trees compared to those in northern forests of either type (Table 3). Links between climate and parenchyma structure could help explain why *Sequoia* trees in secondary forests on relatively dry eastern and southern range margins have higher heartwood extractive content than those in the north^31^, but ray anatomy of trees in marginal secondary forests remains to be explored.

Mechanisms for environmental responsiveness of ray parenchyma development must involve hormonal signals at the level of the cambium, directing formerly fusiform tracheid initials to segment and initiate rays or terminate ray lines^10,32^. The reasons for seasonal effects of rainfall on ray parenchyma development may relate to ethylene and auxin ratios in the cambium, where ethylene induces new rays when concentrated in cambial initials^10^. Ethylene might accumulate in the cambium in response to external factors such as heat, biotic perturbation, low root oxygen, or physical trauma^33–35^, thereby shifting individual cambial initials to ray production. Ethylene presumably promotes development of segmented ray parenchyma initials by suppressing the polar transport of auxin that signals tracheid differentiation^10,32^. Ethylene induces cellular subdivision and prolonged, slow expansion resulting in axially short cells with a long radial orientation^10,36^. Relatively high levels of ethylene and low levels of auxin at the heartwood/sapwood transition zone are also associated with heartwood deposition across many species^37^, heartwood deposition can be increased through ethylene application (Lui et al*.* 2022)^38^and wounding that prompts endogenous ethylene production^39–46^, both of which induce the formation of new ray parenchyma lines^10^. Thus, when ethylene is high in the trunk, we might expect both more rays and more heartwood deposition. How hormonal signals relate to the fate of new rays as short or tall is not immediately apparent, but ethylene is involved in regulating subdivision of fusiform initial into more or fewer ray initials. Intriguingly, when very young, *Pinus* species have only short rays and then gradually, over the first decades of their lives, add tall rays^10,11^. Considering the similar-length delay before initial heartwood formation in many species, and their related hormonal control, ray development may be ontogenetically coupled with heartwood deposition.

Differences in cambial age between trees in primary and secondary forests could be driving differences in parenchyma ray size and type distribution. Accordingly, heartwood deposition may be related to a late ontogenetic shift in this long-lived tree species, possibly linked to whole-tree structural development. In *Sequoia*, short ray density is the most important predictor of heartwood extractive content, but the density of short parenchyma rays decreases with increasing cambial age in secondary forests (Table 3). This is not the case in either northern or southern primary forests, whose older trees lack any of the anatomical correlations with cambial age that are evident in northern secondary forests (Table 3). As tall *Sequoia* develop, they inevitably lose their original tops to disturbance, disrupting apical dominance and promoting initiation of potentially hundreds of trunk reiterations during millennial lifespans^42^. Loss of a central leader may alter whole-tree auxin dynamics in unpredictable ways, possibly limiting the predictability of ray development based on age or crown position. A decrease in short ray density with age in northern secondary forest trees, which still have intact tops, does not necessarily suggest a decline in their heartwood production capacity. Taller and older trees of primary forests have more complicated relationships between anatomy and heartwood deposition (Table 4). Despite substantially higher heartwood extractive contents in rays of primary forest trees (See Fig. 2 and^15^), northern secondary forest trees have ~ 20% more short rays (Table 3), suggesting changes in heartwood deposition as trees grow taller and develop trunk reiterations. Regardless, fewer short rays and taller tall rays as the cambium ages corresponds with observations in *Pinus* of a shift from short-ray-only wood to wood with increasingly tall rays during the first 16 years of tree growth^11^ all before these trees began producing substantial heartwood. Understanding the difference in heartwood development capacity between primary and secondary forests requires more research into the functional significance and interactive effects of the two uniseriate parenchyma ray-types in conifer wood as well as hormonal shifts associated with the development of crown structural complexity.

## Conclusions

The size and abundance of short and tall types of ray parenchyma in *Sequoia* sapwood interact to predict heartwood extractive content (Table 4) with differences in X-ray absorption (Fig. 2) suggesting these ray types have divergent roles in sapwood and heartwood. Future work on the functional significance of the two ray types could reveal trait-based tools to monitor the heartwood responses of *Sequoia* and other species to disturbances, climatic variation, and experimental treatments. Ray development reflects a lasting structural commitment to a particular carbohydrate allocation and heartwood deposition strategy. Sapwood-ray-anatomy models for heartwood extractive content are especially promising for future tool development in actively managed secondary *Sequoia* forests. Heartwood extractives enhance the decay resistance of tree biomass, increasing the length of time that the carbon trees assimilate is stored in forest ecosystems. Forest management designed to promote carbon storage and extend its residency time would benefit from strategies that stimulate heartwood deposition, prioritizing the inaccessible carbon pool that promotes tree longevity over the accessible non-structural carbohydrates in sapwood that support growth or recovery. Determining if feasible management strategies exist could involve assessing anatomical differences among sites and monitoring ray parenchyma development in response to silvicultural treatments. Additional work on climatic and age-related influences on ray-type distribution as well as analysis of their contents will help clarify their functional roles. Anatomy remains one of the clearest indicators we have of plant performance and environmental acclimation. Cell structure and tissue distribution represent binding strategic moves on the part of the plant that last until the decomposition of the cell. In the case of *Sequoia* and other long-lived species, impacts of ray phenotypic plasticity span millennia and deserve further consideration.

## Supplementary Information

Supplementary Information.

## Data Availability

Data and CT images are available on request from the authors.
