# Supplementary material for: Interaction of two parenchyma ray types regulates redwood heartwood deposition
Source: Sci Rep. 2026 Mar 31;16:10847. doi: 10.1038/s41598-026-42938-6 (PMC13039502; doi:10.1038/s41598-026-42938-6)
Supplement: Supplementary file 1 — Supplementary Information. [file 41598_2026_42938_MOESM1_ESM.docx]

**Supplemental**

**Table S1.** Each of our 81 study trees provided a sapwood/heartwood pair for analysis, individual trees were randomly selected from those in Sillett *et al.* 2022 and the site numbers match those in that paper, which also provides maps and further location detail. Precise tree locations are confidential but latitude and landownership information are provided: USFS = US Forest Service, CSP = California State Parks, NPS = National Park Service, P = private, Arcata = City of Arcata, BLM = Bureau of Land Management, LTNC = Land Trust of Napa County, MCP = Marin County Parks, EBRPD = East Bay Regional Park District, UC = University of CA. Forest type refers to primary (P) and previously-logged secondary (S).

Citation:

Sillett, S.C., Antoine, M.E., Carroll, A.L., Graham, M.E., Chin, A.R. and Van Pelt, R., 2022. Rangewide climatic sensitivities and non-timber values of tall Sequoia sempervirens forests. Forest Ecology and Management, 526, p.120573.
